# A unified ensemble soil moisture dataset across the continental United States

**DOI:** 10.1038/s41597-025-04657-x

**Published:** 2025-04-01

**Authors:** Lingcheng Li, Xinming Lin, Yilin Fang, Z. Jason Hou, L. Ruby Leung, Yaoping Wang, Jiafu Mao, Yaping Xu, Elias Massoud, Mingjie Shi

**Affiliations:** 1 Pacific Northwest National Laboratory, 902 Battelle Blvd, Richland, WA 99354 United States; 2 Environmental Sciences Division and Climate Change Science Institute, Oak Ridge National Laboratory, 1 Bethel Valley Rd, Oak Ridge, TN 37830 United States; 3 Department of Environmental and Geosciences, Sam Houston State University, Huntsville, TX 77340 United States; 4 Integrated Computational Earth Sciences group, Oak Ridge National Laboratory, 1 Bethel Valley Rd, Oak Ridge, TN 37830 United States

**Keywords:** Hydrology, Hydrology

## Abstract

A unified ensemble soil moisture (SM) package has been developed over the Continental United States (CONUS). The data package includes 19 products from land surface models, remote sensing, reanalysis, and machine learning models. All datasets are unified to a 0.25-degree and monthly spatiotemporal resolution, providing a comprehensive view of surface SM dynamics. The statistical analysis of the datasets leverages the Koppen-Geiger Climate Classification to explore surface SM’s spatiotemporal variabilities. The extracted SM characteristics highlight distinct patterns, with the western CONUS showing larger coefficient of variation values and the eastern CONUS exhibiting higher SM values. Remote sensing datasets tend to be drier, while reanalysis products present wetter conditions. *In-situ* SM observations serve as the basis for wavelet power spectrum analyses to explain discrepancies in temporal scales across datasets facilitating daily SM records. This study provides a comprehensive soil moisture data package and an analysis framework that can be used for Earth system model evaluations and uncertainty quantification, quantifying drought impacts and land–atmosphere interactions and making recommendations for drought response planning.

## Background & Summary

Soil moisture impacts plant transpiration and photosynthesis, affects the water, energy, and the biogeochemical cycles, and contributes to precipitation and radiation anomalies^1^; it is essential to the carbon cycle and land–atmosphere interactions at various scales and substantially modulates regional climate change^1–3^. Although soil moisture is crucial within the entire Earth system, there is a lack of data coverage and consistent resolution across both time and space, as well as deficiency in monitoring standards pertaining to soil moisture^4^. This deficiency hinders our ability to make informed decisions regarding agriculture, water resource management, climate change mitigation, and ecosystem preservation. Therefore, studying soil moisture and establishing a comprehensive soil moisture database at various spatial and temporal scales, such as the sub-seasonal to seasonal time scales and the regional and continental spatial scales, are essential to environmental science, climate research, and the management of land and water resources ^5–7^.

Soil moisture products derived from various sources offer distinct strengths and limitations, reflecting their diverse applications and methodologies^8^. *In-situ* soil moisture sensors offer real-time data but are at limited spatial scales. Remote sensing products of soil moisture provide global coverages, but some products have relatively coarse spatiotemporal resolutions as a result of instruments’ design and revisit periods (e.g., the Soil Moisture and Ocean Salinity [SMOS]). In addition, passive microwave radiometers (e.g., Soil Moisture Active Passive [SMAP] and SMOS) are not able to obtain sufficient information of soil moisture under dense forest canopies^9,10^. For example, the SMAP and other satellite data products are designed to penetrate only a few centimeters (e.g., 0–5 cm) into the soil surface, measuring moisture at shallow depths. Numerical models and reanalysis datasets offer long-term soil moisture data products, yet they could have considerable uncertainties resulting from the meteorological forcing, model input data characterizing the surface features (e.g., soil texture, topography), and model’s physical representations^11,12^. Recent CMIP soil moisture inter-comparison studies^12,13^ revealed discrepancies and limitations in soil moisture data across various ecosystems, highlighting the need for improved modeling strategies. In general, using machine learning (ML) models can reasonably capture the spatiotemporal variations of soil moisture, whereas the data accuracy can be limited due to soil moisture sampling density across regions^14^. That is the accuracy of the ML data is closely related to the site density of *in-situ* soil moisture measurements that are the target variable of ML models. Consequently, these diverse data sources often generate disparate results due to the accuracy and uncertainty from both measurements and models. Exploring the strengths and limitations of various soil moisture products becomes increasingly imperative, necessitating continued interdisciplinary efforts to enhance the accuracy and comprehensiveness of soil moisture representations and predictions across different scales and environmental settings.

Previous studies reveal limitations in terms of measurement and modeling capacities, as highlighted above, resulting in an incomplete understanding of soil moisture dynamics. In addition, the integration of diverse soil moisture datasets poses challenges due to the differences in measurement techniques, scales, and resolutions. To harness the full potential of available soil moisture information, mitigate their limitations, and unlock the synergistic potential of multi-source datasets, it is essential to compare and integrate data from multiple sources through the development of standardized protocols and establishment of inclusive soil moisture data packages. These data packages can be ensured to have reasonable spatial and temporal coverages based on the community’s needs, quantification of data uncertainty, and comprehensive data validation, and therefore promote better understanding and effective monitoring of soil moisture dynamics. In addition, these data packages can largely benefit data assimilation, machine learning model training, and serve as the foundation for informed decision-making and sustainable environmental stewardship^15^. Currently, there is a lack of comprehensive datasets and data intercomparison framework that adequately address these challenges for soil moisture.

This study aims to bridge the knowledge gaps in understanding soil moisture discrepancies among different products and enhance the assessment of soil moisture accuracy and reliability. We select the Continental United States (CONUS) as our research area, which is a region facing an increase in extreme weather events and their impacts^16^. We are motivated to recompile soil moisture from a variety of sources and explore soil moisture features in various sub-domains of CONUS by comprehensively integrating and assessing soil moisture from a variety of sources. We examine the variability of soil moisture across different spatiotemporal resolutions and quantify the associated uncertainties, through which we address the intricate balance between the strengths and limitations of different soil moisture products, ensuring that the data accurately represent the dynamics of soil moisture in various contexts. By doing so, we seek to provide insights into the factors contributing to soil moisture discrepancies and identify opportunities for improvement of data products. Through the utilization of this database, the community can have a substantial understanding of the soil moisture features, the impacts of the water cycle on the carbon and nutrient cycles, and the land–atmosphere feedback over CONUS. The data products, together with our rigorous analysis, provided by this research will ultimately guide data users in scientific inquiries and facilitate more robust model–data comparisons across scales. Last but not the least, this database can find broad applications in aiding precision agriculture, hydrology, climate modeling, and disaster management.

## Methods

### Gridded soil moisture data sources

We gathered both *in-situ* soil moisture measurements and a comprehensive collection of gridded soil moisture data products to evaluate the data variability and quantify the data uncertainty over CONUS. The *in-situ* soil moisture data is from the international soil moisture network (ISMN)^17,18^, while the gridded soil moisture data include four categories: land surface model (LSM), remote sensing (RS), reanalysis (RE), and machine learning (ML) products (Table 1). By using data characterizing the meteorological and surface conditions and solving water and energy balance equations, LSMs simulate soil moisture in different soil layers, and can be used to develop spatially- and temporally- continued records with vertical profiles. RS uses microwave or thermal sensor measurements to estimate soil moisture from space and provides invaluable global coverages. RS has limitations in characterizing soil moisture diurnal cycles, because satellites usually have the revisit time of a certain location in days. For example, SMAP^19,20^ and SMOS^21^ provide a global soil moisture estimate every 2–3 days. RE products are developed through assimilating satellite and ground observations with certain assimilation schemes, and they are gap filled soil moisture products with vertical profiles^6^. ML algorithms enable the extraction of high-resolution soil moisture information from point observations and a variety of data sources (e.g., remote sensing, surface features), while enhancing data accuracy^22^ (e.g., Han *et al*.^22^). Thus, the gridded data collection encompasses a multifaceted approach that leverages diverse techniques, and the synergy between these methods empowers researchers to construct comprehensive soil moisture datasets that facilitate critical scientific insights and applications across scales.

**Table 1:** The summary of the gridded soil moisture data products collected in this study.

Index#	Data Type	Name	Spatial resolution	Temporal resolution	Vertical distribution	Time span	Data sources
1	LSM	LSM_GLDAS_NOAH	0.25 deg	3-hourly	Surface to 200 cm	2000–present	Rodell *et al*.^24^
2		LSM_NLDAS_MOS	0.125 deg	hourly	Surface to 200 cm	1979–present	Schaake *et al*.^25^
3		LSM_NLDAS_NOAH	0.125 deg	hourly	Surface to 200 cm	1979–present	Schaake *et al*.^25^
4		LSM_NLDAS_VIC	0.125 deg	hourly	Surface to 200 cm	1979–present	Schaake *et al*.^25^
5	RS	RS_ESA_CCI	0.25 deg	Daily	Surface	1978–2021	Dorigo *et al*.^32^
6		RS_MCCA	0.25 deg	Daily	Surface	2002–2021	Hu *et al*.^35^
7		RS_SMAP_L3	9 km	Daily	Surface	2015–present	O’Neill *et al*.^21^
8		RS_SMOS_L3	25 km	Daily	Surface	2010–present	Kerr *et al*.^19^
9	RE	RE_ERA5_Land	9 km	Hourly	Surface to 289 cm	1950–present	Balsamo *et al*.^56^
10		RE_GLEAM	0.25 deg	Daily	Surface & root zone**	2003–2021	Martens *et al*.^57^
11		RE_SMAP_L4	9 km	Daily	Surface & root zone**	2015–present	Reichle *et al*.^58^
12		RE_GDSMFD	25 km	Daily	Surface to 100 cm	2011–2018	Xie *et al*.^59^
13	ML	ML_CASM	25 km	3-day	Surface	2002–2020	Skulovich & Gentine^60^
14		ML_GSSM	1 km	Daily	Surface	2000–2020	Han *et al*.^22^
15		ML_SoMo	0.25 deg	Daily	Surface to 50 cm	2000–2019	Sungmin & Rene^31^
16		ML_GLASS	1 km	Daily	Surface	2000–2020	Zhang *et al*.^14^
17		ML_ZHENG23*	1 km	Monthly	Surface	2000–2020	Zheng *et al*.^61^
18		ML_NNsm	36 km	Daily	Surface	2002–2021	Yao *et al*.^36^
19		ML_RSSSM	0.1 deg	10-day	Surface	2003–2018	Chen *et al*.^62^

*For any of the dataset without a name in the description paper, we name the data with the first author’s name and the data publishing year. For example, ML data product by Zheng *et al*.^61^ is named as “ML_ZHENG23”

**root zone represents the 0–100 cm depth^56,57^.

In this study, 19 gridded soil moisture datasets from the above-mentioned four data categories were collected, and they include four LSM products, where NLDAS provides soil moisture products from three different LSMs, four RS products, four RE products, and seven ML products^23^ (Table 1). Each dataset has a dedicated repository, and we follow the data use policy and data download procedures of each data set to retrieve and archive the data. Here, each data category includes more than four products, providing a sufficient representation of the general features of each data type.

For the data selection, we comprehensively consider different factors, including time spans and the spatial and temporal resolutions. Specifically, for the LSM data selection, we use the data from the Global Land Data Assimilation System (GLDAS)^24^ and the North America Global Land Data Assimilation System (NLDAS)^25^. Compared to the land model outputs from the Coupled Model Intercomparison Project (CMIP)^26^, which are at the 1–2 degree spatial resolutions, both GLDAS and NLDAS have a relatively higher spatial resolution, 1/8 degree. In addition, the CMIP based intermodal comparison of soil moisture is out of the scope of this study. We also try to integrate RE datasets with relatively higher spatial resolution in our data package. For example, the Modern-Era Retrospective Analysis for Research and Applications Version 2 (MERRA) and the Japanese 55-year Reanalysis (JRA-55) are at the 0.5° × 0.625° and 1.25° × 1.25° resolutions, respectively^27^; thus, we use the ERA-5 Land data, which are at the 9 km spatial resolution. When we were selecting the ML datasets, the temporal coverage is the primary factor, since there are many soil moisture datasets only have several years of data coverage (e.g., Huang *et al*.; Long *et al*.^28,29^). All the ML data in this study have the coverages longer than 15 years, which can accommodate with the temporal coverage of other datasets.

Different from other soil moisture data sources that are generated with process-based or statistical ML models, RS soil moisture data are based on single- or multi-sensor passive microwave measurements applied to different data retrieval algorithms^10,30^. In this study, we choose to use RS soil moisture from four different sources, and they are SMAP, SMOS (from the Centre Aval de Traitement des Données SMOS (CATDS)), the Climate Change Initiative of the European Space Agency (ESA CCI), and the multi-channel collaborative algorithm (MCCA) based on the inter-calibrated AMSR-E/2 multi-frequency passive microwave measurements (Table 1). To improve the data accuracy and quality, different research groups could update the RS soil moisture data by improving the representations of spatial heterogeneity and using multi-temporal and multi-angular retrieval approaches^31^. Here, we use the SMAP and SMOS L3 soil moisture products developed from the original retrieval algorithms of these two missions, which rely on the single-channel and multi-angular observations, respectively^30^. These choices reveal the data features based on the original instrument and algorithm development, and comparing different data versions based on measurements from the same instrument is beyond the scope of this study. The ESA CCI soil moisture datasets are the merged soil moisture retrievals from a series of microwave sensor systems, and contain active, passive, and combined sets. It shows that the combined product performs best among the three sets, so we use the ESA CCI combined set in our study^32,33^. This study performs statistical analysis of soil moisture of different types, so we use the AMSR2 MCCA (the operation of AMSR-E stopped in 2011) soil moisture as another RS soil moisture product. Since AMSR2 retrievals may not always reliably represent soil moisture content deeper than approximately 1 cm from the surface, they tend to show dry biases when compared to *in situ* measurements taken at a depth of 3 cm in semi-arid areas^34^. Therefore, we choose to use AMSR2 MCCA, which demonstrates good performance when validated against soil moisture networks (with the shallowest layers being <5 cm)^35^.

### Soil moisture data processing and integration

The soil moisture products show varying spatial-temporal resolutions, with the spatial resolution ranging from 1 km (e.g., ML_GSSM^22^) to 36 km (i.e., ML_NNsm^36^), and the temporal resolutions ranging from hourly to monthly (Table 1). Among all the 19 datasets, 8 datasets are at the 0.25 degree/25 km spatial resolution. To minimize errors induced by data interpolation, we perform data comparison at the 0.25 degree spatial resolution. All datasets that are not at the 0.25 degree spatial resolution are remapped by using the nearest neighbor method for downsampling and the averaging method for upsampling, respectively. In other words, data products with finer spatial resolutions (e.g., 0.125°) are upscaled to 0.25° using the averaging method, while the dataset with coarser spatial resolutions (i.e., 36 km) are downscaled to 0.25° using the nearest neighbor resampling method. For all the RS datasets with ascending and descending tracks, we calculate the ascending and descending average to represent the mean soil moisture status. RS and ML products do not have vertical profiles of soil moisture, so we perform data analyses for the surface layer (i.e., 0–5 cm depth; Table 1).

All the data products are averaged to the monthly temporal resolution, and we also process the data to the daily time scale (except for three products with coarse temporal resolutions; Table 1) for wavelet analysis, which provides an enhanced understanding of temporal variations of the datasets. Since the daily map of RS datasets cannot have a full coverage of the globe, averaging the ascending and descending tracks will further reduce the data coverage (e.g., setting the unobserved areas with missing values). Given that remote-sensing descending retrievals are found to have better agreement with *in-situ* measurements^37^, we use the soil moisture retrievals based on descending observations to represent soil moisture status at the daily time scale. For datasets with finer temporal resolutions (e.g., hourly, 3-houly), we calculate the arithmetic means to obtain the daily records.

The gridded soil moisture products also have various temporal coverages, and the common coverage period is 2016–2018. For example, the Soil Moisture Active Passive (SMAP) mission was launched in April 2015, with data available from April 2015 to 2023, and the RS_GDSMFD has the coverage period of 2011–2018 (Table 1). Thus, to make use of all the datasets, we perform the data variability evaluation and uncertainty quantification during 2016–2018. Extending the data time frame to 2016–2020 reduces the total number of datasets to 16. We apply the same data analysis methods for evaluating variability and quantifying uncertainty across both time periods, and the results do not affect our overall conclusions (Technical Validation). Since RS datasets are largely affected by snow covers in the cold season^38^, i.e., November–March, we use all the data records from April to October to calculate the arithmetic mean and perform the data analysis. The all-season soil moisture features are discussed in Text S1 and shown in Fig. S2.

**Fig. 1:**
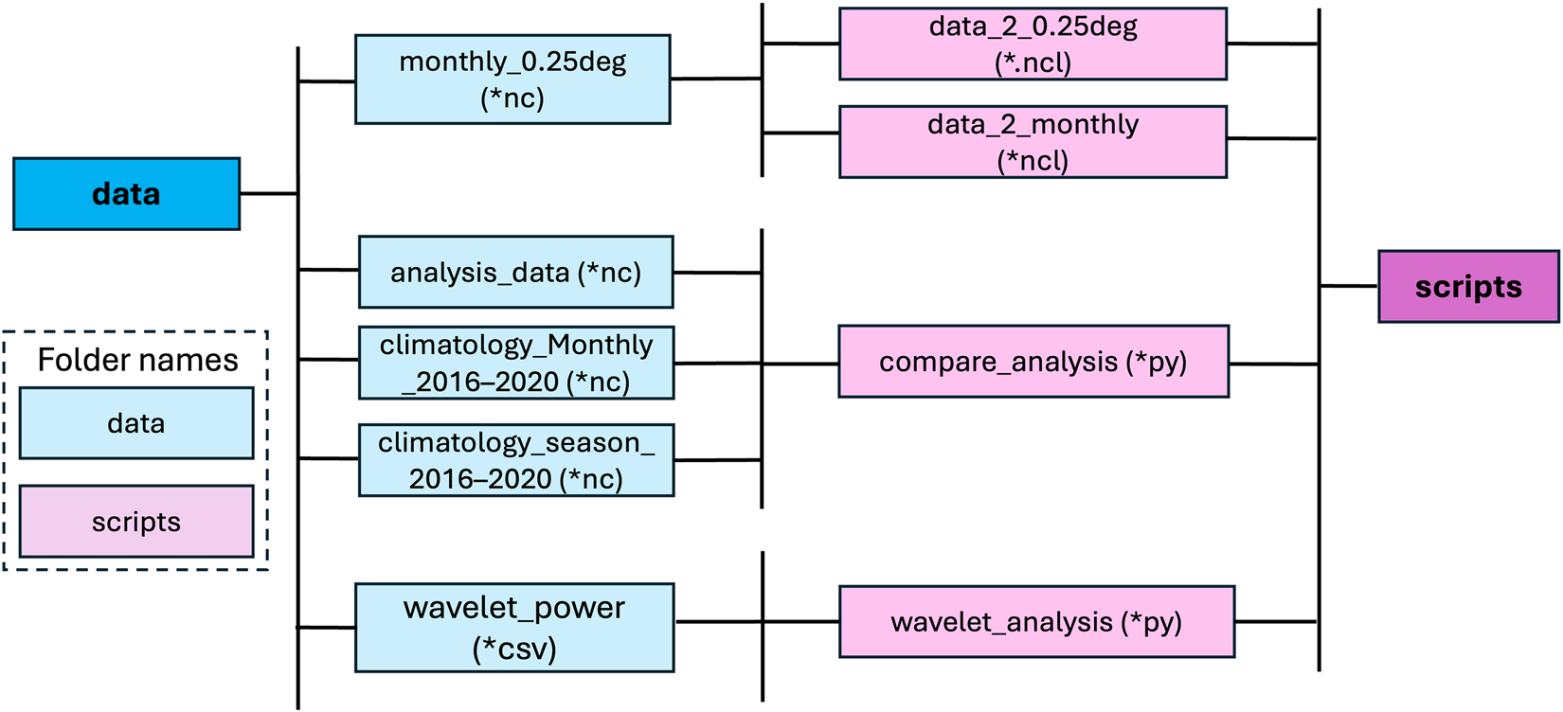
The “data” and “script” folders and their connections in the Ensemble Unified Soil Moisture (EnUSM) data package. “nc” represents Netcdf, “py” represents Python, “ncl” represents NCAR Command Language, and “csv” represents Comma Separated Values.

We explore the soil moisture spatial variations in six Köppen-Geiger climate classifications (KGCCs) over CONUS^39^ (*Auxiliary Datasets*; Fig. 1a). To have a comprehensive understanding of the features of the data, we compute the April–October mean soil moisture of all the datasets across 2016–2018 (Fig. 1b). We also perform data analysis in different KGCCs of different data types to investigate the data variability between data types (Fig. 1c).

### Auxiliary datasets for soil moisture characterization

With the compiled data sets, we conduct an extensive variability analysis to extract the spatiotemporal features of soil moisture across various seasons and regions over CONUS. Here, we use the KGCC map^39^, which is upscaled from 0.0083° to 0.25° spatial resolution through a majority sampling method, to study the soil moisture features across CONUS. CONUS includes six major KGCCs, and they are: Arid Desert (BWh, BWk), Arid Steppe (BSh, BSk), Cold DrySum (Dsa, Dsd), Temperate DrySum (Csa, Csc), Cold NoDry (Dfa, Dfd), and Temperate NoDry (Cfa, Cfc) (Fig. 1a).

To illustrate the spatial patterns of soil moisture characteristics, we use datasets characterizing the environmental conditions such as annual mean precipitation and temperature based on the 2016–2018 Multi-Source Weather (MSWX) dataset^40^, and the aridity index^41^. Soil moisture dynamics are also regulated by the interactions between climate conditions and the characteristics of soil and vegetation. For example, soil properties influence soil water retention, infiltration rates and surface runoff. Topographical features influence the runoff pathways, hence the spatial distribution of soil moisture. Vegetation characteristics, such as leaf area index (LAI), can influence interception, transpiration, and the partitioning of rainfall. Thus, we also use data variables from the global 1 km land surface datasets^42^, which include soil properties (i.e., sand percentage), topographical features (i.e., elevation, standard deviation of elevation, and slope), and LAI. In addition, the contributions of distance to the nearest coast (Dist; generated by NASA’s Ocean Biology Processing Group, https://oceancolor.gsfc.nasa.gov/resources/docs/distfromcoast/) to soil moisture spatial features are also assessed. Here, the values of Dist range from negative to zero, moving from inland towards the coast, where regions further inland exhibit more negative values. All these datasets are remapped to the 0.25 degree spatial resolution.

### Extracting influential factors on soil moisture

To investigate the factors determining the spatial features of soil moisture, including mean values, standard deviation, coefficient of variation, and discrepancies across different datasets, we adopt the eXplainable Machine Learning (XML) framework. The machine learning algorithm is eXtreme Gradient Boosting (XGBoost^43^). XGBoost is a decision-tree-based ensemble learning model adept at handling complex variable interactions and collinearity, making it invaluable for addressing Earth science research. Its versatility is demonstrated in diverse applications such as aerosol property estimation^44^ and parameter sensitivity analysis^45^.

The explainable variables for XGBoost model training include climate conditions, soil properties, topographical features, and LAI (*Auxiliary Datasets*). Thus, the XGBoost model is equipped with a comprehensive package that mirrors the complex nature of soil moisture patterns. The training of the XGBoost model is a rigorous process, emphasizing hyperparameter optimization through Bayesian methods^46^ and employing five-fold cross-validation to effectively reduce the risk of overfitting. The optimization focuses on minimizing mean squared error, with the model’s performance being assessed using the R-squared metric.

Based on the trained XGBoost models, we use SHapley Additive exPlanations (SHAP)^47,48^ for an in-depth interpretation of the model’s outputs. SHAP employs a game-theoretic approach to offer a detailed and quantifiable assessment of the significance of each feature, thereby shedding light on how different variables contribute to the observed spatial patterns of soil moisture. By integrating modeling with XGBoost and subsequent explanation through SHAP, this XML strategy facilitates a nuanced understanding of the determinants of soil moisture distribution, justifying the effective merger of machine learning and explanatory analysis in the realm of environmental science.

### Frequency domain characteristics of soil moisture

Wavelet analysis^49^ is a common tool for analyzing time series with many different timescales or changes in variance. The fundamental idea behind wavelets is to analyze according to scale. By decomposing a time series into time-frequency space, it can help determine both the dominant timescales of variability and how those timescales vary in time. The decomposition leads to a good trade-off for the time-scale resolution, which is related to frequency resolution^50^. This can be regarded as a partition of the variance of the series into its different oscillating components with different frequencies (periods). This wavelet transform decomposes time series over wavelet prototype functions called “mother” Morlet wavelet *φ*(*t*)^51^, which is defined as

1 $${\varphi }_{a,\tau }\left(t\right)=\frac{1}{\sqrt{a}}\varphi \left(\frac{t-\tau }{a}\right)$$

where $\tau$ is the time position, and $a$ is the scale of the wavelets. The Morlet wavelet transform of a time series $x(t)$ with respect to a chosen mother wavelet is performed as follows^52^:

2 $${W}_{x}\left(a,\tau \right)=\frac{1}{\sqrt{a}}{\int }_{-\infty }^{+\infty }x\left(t\right){\varphi }^{\ast }\,(\frac{t-\tau }{a})\,{dt}={\int }_{-\infty }^{+\infty }x\,(t\,){\varphi }_{a,\tau }^{\ast }\left(t\right){dt}$$

where * denotes the complex conjugate form (i.e., the imaginary part), the wavelet coefficients, ${W}_{x}\left(a,\tau \right)$ represent the contribution of the scales $a$ to the time series at different time positions $\tau$. The wavelet power spectrum, which has an interpretation of time-period (time-frequency) wavelet energy density, can be calculated using^53^

3 $${{Power}}_{x}\left(a,\tau \right)=\frac{1}{a}{\left|{W}_{x}\left(a,\tau \right)\right|}^{2}$$

As mentioned before, the wavelet decomposition can be regarded as a partition of the variance of the series into its different oscillating components with different periods (frequencies). Peaks in the power spectrum indicate which timescales/frequencies are contributing the most to the variance of the series.

In this study, we use the continuous wavelet transform to decompose the daily soil moisture time series, which includes soil moisture data from *in situ* soil measurements, as well as the LSM, RS, RE, ML data products, to determine the dominant timescales of variability for these time series and how those timescales vary in time at a specific location of the six primary KGCCs (i.e., Arid Desert, Arid Steppe, Cold DrySum, Temperate DrySum, Cold NoDry, and Temperate NoDry). For each KGCC zone, we first choose a station that is not only close to the geographic center of that zone but has relatively complete *in situ* soil measurements (i.e., less than 50% of missing value for each season; Table 2) from 2016 to 2018. Note that the period from 2016 to 2018 is the period with relatively complete daily soil moisture data for most data products. Then continuous wavelet transform is performed for the daily soil moisture time series of the chosen station, as well as the data products with available daily soil moisture data near that chosen station.

**Table 2:** The selected sites for wavelet analysis (Sites are mapped in Fig. 2).

The KGCC of sites	latitude	longitude	Vegetation type	Mean soil moisture (±1 std; m^3^ m^−3^)
Yuma-27-ENE	32.8350°	−114.1884°	Arid Desert	0.088 ± 0.047
Williams-35-NNW	35.7552°	−112.3374°	Arid Steppe	0.107 ± 0.057
TonziRanch	38.4316°	−120.9660°	Temperate DrySum	0.205 ± 0.152
Newton-5-ENE	32.3378°	−89.0703°	Temperate NoDry	0.239 ± 0.086
WATERHOLE	47.9400°	−123.4300°	Cold DrySum	0.186 ± 0.087
Bedford-5-WNW	38.8882°	−86.5707°	Cold NoDry	0.33 ± 0.080

## Data Records

The recompiled ensemble soil moisture data, unified at 0.25 degree and monthly spatiotemporal resolution, is named Ensemble Unified Soil Moisture (EnUSM). The data across 2016–2020 can be obtained from Zenodo https://zenodo.org/records/1454223936. The directory structure is shown in Fig. 1. All gridded data are in the NetCDF format. The *in-situ* soil moisture at the selected six sites (Fig. 1a) and the co-located gridded data are included in Comma-separated value (CSV) files for the wavelet analyses. The *script* folder includes Python and NCAR Command Language (NCL) based scripts for data processing and analysis. We also recompile the 16 soil moisture datasets to a 0.25 degree and daily spatiotemporal resolution. The size of this entire data package is 39 gigabytes. Therefore, instead of publishing the daily soil moisture records on Zenodo, users can request the daily data by contacting our research team.

## Technical Validation

### Statistical validation across koppen-geiger climate classification (KGCCs) of CONUS

The depth of soil moisture varies between different datasets and datatypes, and we perform all the data comparison and evaluation for surface soil moisture (i.e., 0–5 cm) at the 0.25 degree and monthly spatiotemporal resolution. The common time period of the 19 datasets is 2016–2018 (Table 1), and extending the time period to 2016–2020 reduces the dataset number to 16. To be comprehensive, we include the results of 2016–2018 in this Section, and the figures based on 2016–2020 are included in SI.

**Fig. 2:**
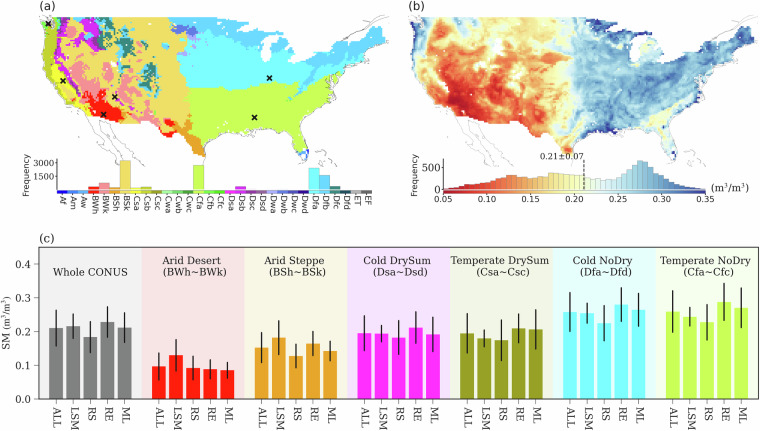
(**a**) KGCC map, sites used in wavelet analysis are marked with “x”; (**b**) mean soil moisture (SM) of all 19 soil moisture (SM) products over the growing season (April–October) during 2016–2018; (**c**) averaged soil moisture for all the 19 datasets (ALL) and for each data type (i.e., LSM, RS, RE, ML) across CONUS (gray bars) and in different KGCCs (colored bars), where error bars show the spatial averages of SM standard deviations across data types. The histograms in (**a,b**) show the frequency distribution of the climate zones and the SM mean of the 19 datasets, respectively.

The arithmetic mean of all the surface soil moisture records from the 19 data sources shows that the dry-to-wet shift of soil moisture from the west to the east (Fig. 2b) displays a similar spatial pattern to that of the KGCC dry-to-wet transition (Fig. 2a). Different data sources suggest various soil moisture features in different KGCCs (Fig. 2). The RS products suggest drier conditions compared to other data types (Fig. 2c). RE datasets are in general wetter than other data types, especially in the “Cold DrySum”, “Cold NoDry”, and “Temperate NoDry” KGCCs. The soil moisture magnitudes of LSM and ML based products are intermediate over CONUS, whereas those from LSM suggest the wettest conditions in the “Arid Desert” and “Arid Steppe” KGCCs (Fig. 2c). All the data types show a relatively small variability in all the “Arid” KGCCs compared to that in other KGCCs. The data variability of LSM products are relatively smaller than that of other products in all the “Cold” and “Temperate” KGCCs, while ML products show relatively smaller variability in all the “Arid” KGCCs, indicating more consistent performance of these data products in certain KGCCs. In all the “NoDry” and “DrySum” KGCCs, the RS, RE, and ML products exhibit more variability within their respective datasets, suggesting higher uncertainties of soil moisture in these KGCCs. The coefficient of variation (CV) of the 19 datasets shows that the soil moisture relative variability has large values in all the “Arid” regions and in Florida, which belongs to the “Temperate NoDry” KGCC but has a relatively dry soil moisture condition (Figs. S1a and 2b). The analysis across 2016–2020 shows quite similar results (Fig. S5).

**Fig. 3:**
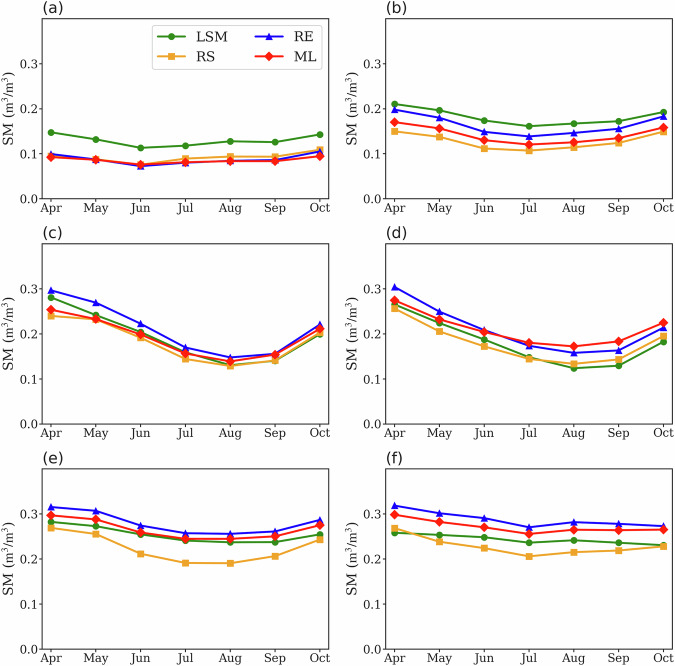
The seasonal cycles for the four types of soil moisture (SM) products during the growing season (April to October) across KGCCs. Each curve represents the aggregated SM mean of a specific data type (i.e., LSM, RS, RE, or ML). The calculation is performed across all the data grids in each KGCCs: (**a**) Arid Desert, (**b**) Arid Steppe, (**c**) Cold DrySum, (**d**) Temperature DrySum, (**e**) Cold NoDry, and (**f**) Temperate NoDry.

The seasonal variability of different data types across KGCCs indicates that the soil moisture seasonal variations are largest in the two “DrySum” KGCCs, with a drier condition in July, August and September than in the other months (Fig. 3c,d). The seasonalities are minimal in the “Arid Desert” and “Temperate NoDry” KGCCs (Figs. 3a,f, and S2). The seasonal variability further confirms that RS products are driest among all data types across KGCCs with the smallest discrepancy between RS, RE, and ML in the “Arid Desert” KGCC (Figs. 2c, 3a), while LSM is the wettest data type in all the “Arid” KGCCs (Fig. 3a,b, S2a, and S2b). The April–October soil moisture seasonalities are similar among datasets, and all the data types show similar seasonality in each KGCC, with RS showing a relatively larger seasonality than other data types in “Cold NoDry”. We also include the seasonal patterns derived from data across 2016–2020 in Fig. S6, with patterns similar to those in Fig. 3. The similar seasonalities among soil moisture data types can also be identified from the normalized soil moisture seasonality (Fig. S3).

**Fig. 4:**
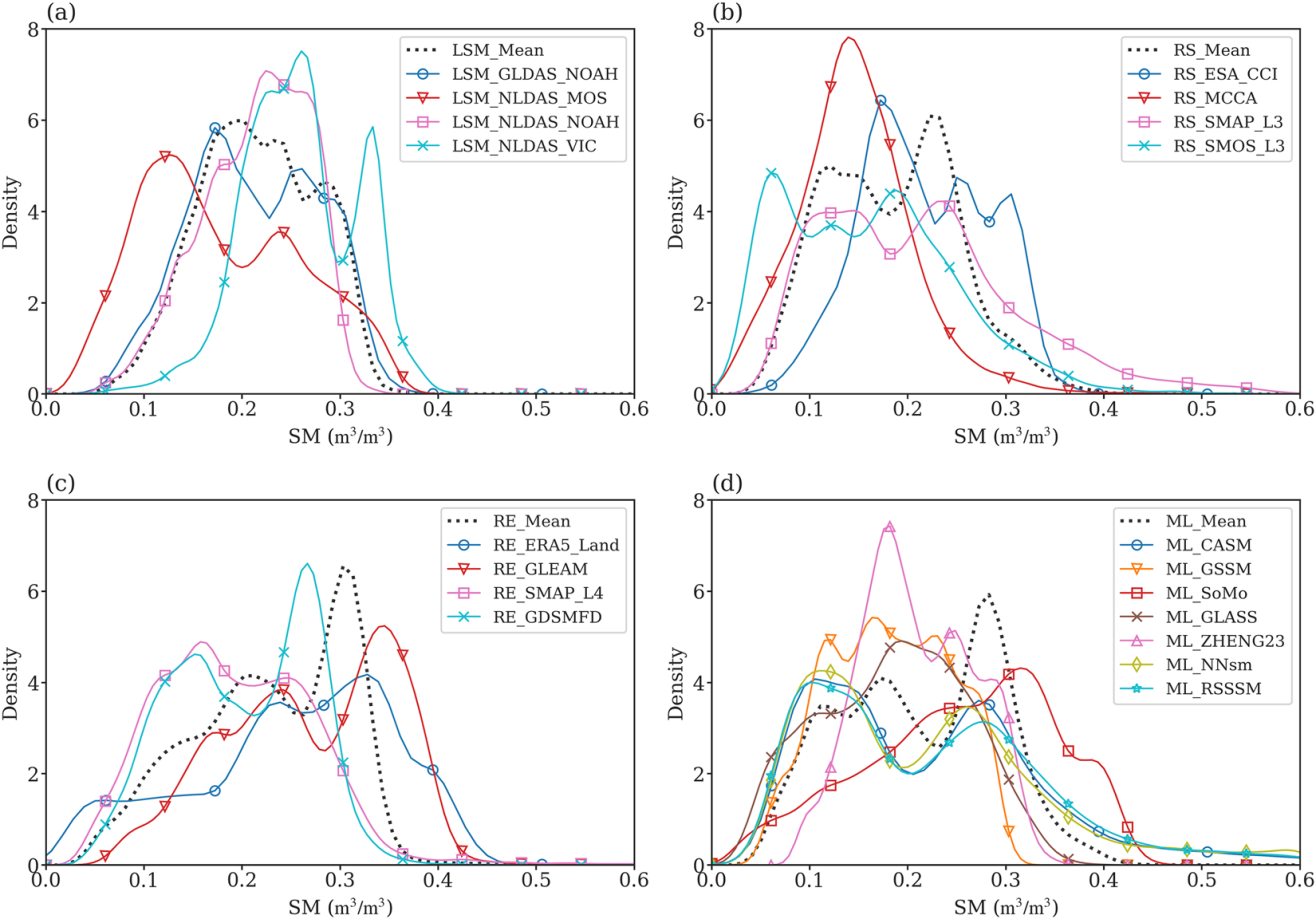
Kernel density estimation (KDE) for the spatial patterns of growing season mean soil moisture (SM) across various products: (**a**) LSM, (**b**) RS, (**c**) RE, and (**d**) ML for the 2016–2018 period. The solid lines represent individual products, while the dashed lines show the aggregate SM mean for each data type.

**Fig. 5:**
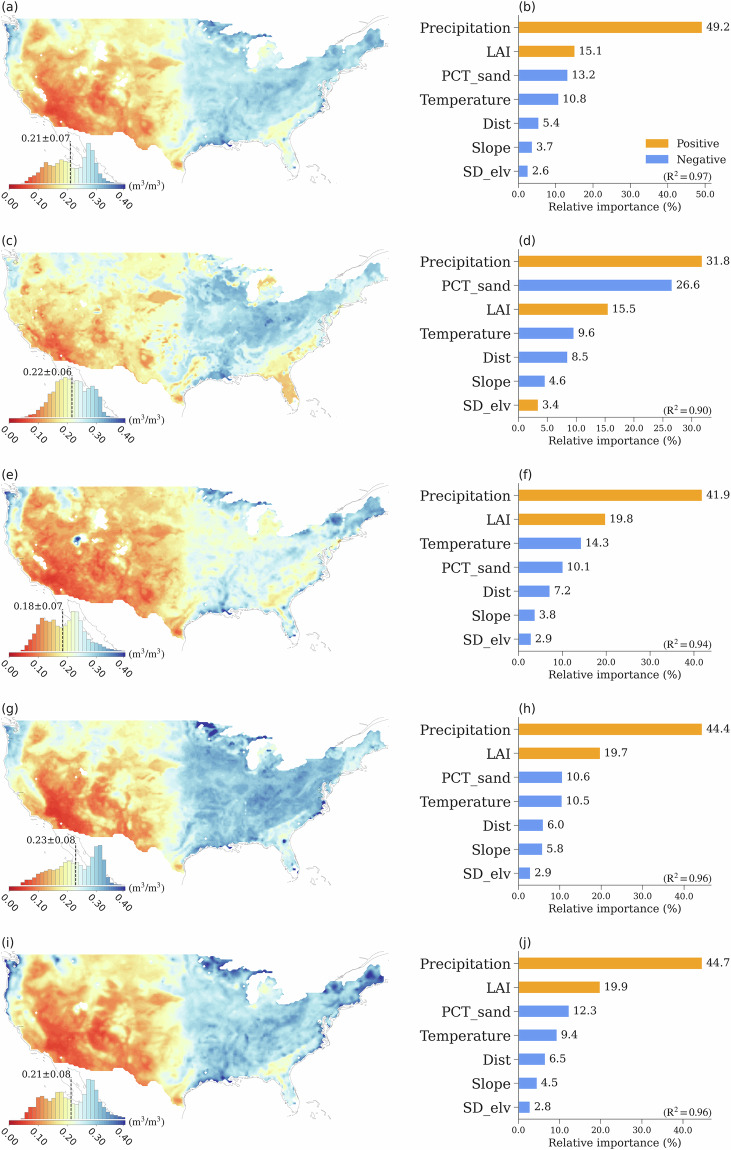
The spatial patterns of mean soil moisture (the left column) and their spatial drivers (the right column), for (**a,b**) all datasets, (**c,d**) LSM, (**e,f**) RS, (**g,h**) RE, and (**i,j**) ML. The histogram plots included in the spatial maps illustrate the probability distribution of the mean soil moisture across CONUS. The feature importance of each variable in determining the spatial variability is calculated as the ratio of the mean |SHAP value| of the variable to the sum of the mean |SHAP value| of all variables. Therefore, the sum of the relative importance of all variables is equal to 100%. SD_elv denotes the standard deviation of elevation, PCT_sand represents soil sand percentage, and Dist represents the distance to the nearest coast. Details of the data sources are provided in the Methods section.

**Fig. 6:**
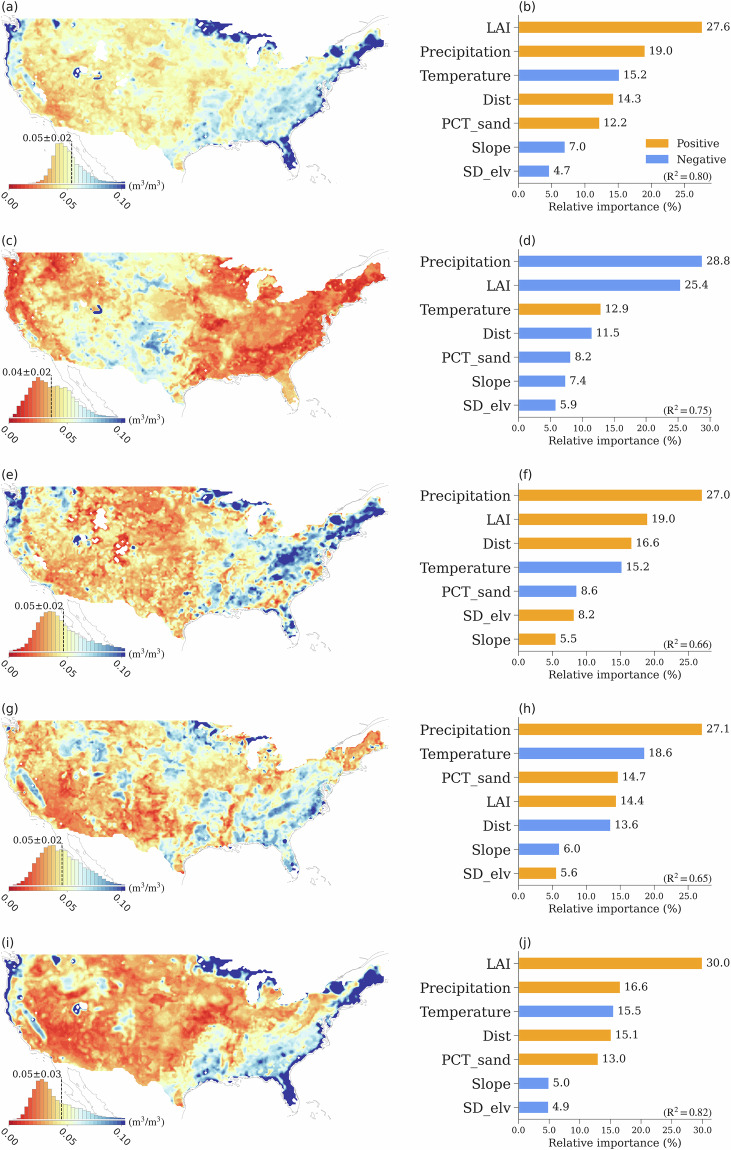
Same as Fig. 5 but for the standard deviation (SD) of soil moisture among data products of the same types. Dist is the distance to the nearest coast. PCT_sand denotes the percentage of sand in the soil. Slope refers to the topographic gradient, derived from elevation data. SD_elv is the standard deviation of elevation. Details of the data sources are provided in the Methods section.

We apply the kernel density estimation (KDE) to soil moisture spatial patterns that are obtained from the growing season mean to interpret soil moisture spatial distribution through estimating the probability density function (PDF) across different data types. Of all the KDE groups, the aggregated KDE of the combined LSM data exhibits a more uniform distribution, indicating that the soil moisture values of LSMs are statistically more consistent compared to other datasets (Fig. 4a). The KDEs of RS indicate that the KDEs of SMAP and SMOS have flatter distributions compared to the other two RS products, implying that the SMAP and SMOS soil moisture exhibits greater statistical uniformity. Among all the KDE curves of the RS dataset, the KDE shape of MCCA is steeper than that of other data products, indicating that the soil moisture values are concentrated in a relatively narrower band. This KDE pattern is consistent with the spatial distribution of Fig. S4f. For the RE and ML data types, the KDEs of the soil moisture means across datasets suggest a high-density value, ~0.3 m^3^ m^−3^,  and they are consistent with the CONUS mean soil moisture features represented by these two data types (Fig. 5g,i). The interquartile range of multi-data mean of RE and ML are wider than that of LSM and RS, implying relatively larger spatial variabilities in soil moisture for RE and ML. The KDEs of the soil moisture means of RS, RE, and ML show multimodal distributions, implying distinct modes of soil moisture due to surface features, such as soil properties, topography, climate, and vegetation cover. Given the substantial variability observed in the KDEs, there is a need to characterize surface features and climate to investigate the contributions of different environmental features in determining the soil moisture spatial distributions (Figs. 5, 6). Note that similar conclusions can be drawn from Fig. S7 for the 2016–2020 period.

### The effects of environmental factors on soil moisture

In this study, we use eXplainable Machine Learning (XML, see Methods) to assess the feature importance of various environmental factors in determining the spatial features of different soil moisture product types. Here, we still use the monthly soil moisture data records of all the 19 datasets. Consistent with the KGCC based analysis for all datasets (Fig. 2c), the spatial maps of different soil moisture data types show the driest condition of RS soil moisture among the four data types (Fig. 5). The dry-to-wet shifts represented by the four data types follow the KGCC indicated dry-to-wet transition. In all the “Arid” and “Dry” KGCCs, LSM products suggest relatively wetter conditions than other data types, while RE products are the wettest among all the “NoDry” KGCCs (Figs. 2a, 3, 5).

The feature importance analysis of SM indicates that precipitation, leaf area index (LAI), surface air temperature, and percentage of sand are the primary factors determining the spatial distribution of the 19-data mean. For all the datasets and each dataset group, the total contributions of these four factors to soil moisture spatial variability are ~80%. The percentage of sand plays an essential (second most important) role in characterizing the LSM soil moisture, revealing the contribution of soil texture in the governing equations in land surface models (e.g., the Noah model), and it is also the fourth most important factor for other soil moisture types. The total contribution from slope, standard deviation of elevation (SD_elv), and distance to coast (Dist) ranges from 12.2% of RS to 19.1% of RE. All these three factors have a negative relationship with soil moisture, with the exception of SD_elv in LSMs.

The standard deviation values among data products of the same type, in general, are smaller in the western CONUS than those in the eastern CONUS, where their spatial distributions are similar for RS, RE, and ML (Fig. 6). LSM products have much smaller standard deviation values compared to other data products. The largest standard deviation values among the LSM products are in the central regions of the CONUS, which is in stark contrast compared to other data types where larger standard deviation values are usually found in the coastal regions in the western and eastern CONUS. ML products have the largest standard deviation values over the eastern coastal regions, primarily due to the relatively large soil moisture values from the ML_RSSM, ML_CASM and ML_NNsm products (Figs. S4m, S4r, and S4s). A similar pattern is also captured by the RS products, where the SMAP product is the primary reason for the large standard deviation values (Fig. S4g). The five-year (2016–2020) mean of each dataset is shown in Fig. S8, with the soil moisture spatial patterns remaining unaffected by the change in time period.

We also use the same XML framework to investigate the importance of the same environmental factors to the standard deviation of soil moisture across data products of the same type. The importance of features in explaining the spatial variation of the standard deviation of soil moisture differs significantly among different data groups. Compared to the importance of the factor variations in explaining the soil moisture means, the importance of various factors with the same data group differs more in explaining the spatial variation of standard deviations. For the standard deviation of all the 19 data products, the most important factors are precipitation, LAI, distance to coast, surface air temperature, and PCT_sand with a total contribution of 88.3% in explaining the spatial variations (Fig. 6b). This feature importance is broadly consistent with that represented by RS and ML (Fig. 6b,f, and j). In general, precipitation, LAI, and distance to coast are positive factors to spatial variability of soil moisture standard deviation of different types. For LSMs (Fig. 6c and d), the impact of these three factors appears to be opposite in direction, with only surface air temperature exhibiting a positive influence in predicting soil moisture standard deviations among data products. This notable discrepancy in LSM-derived soil moisture, especially in arid regions, may stem from the intricate modeling of soil moisture dynamics in LSMs. LSMs include a range of processes such as vegetation transpiration, soil hydraulics, groundwater dynamics, and runoff, all of which contribute to the complexity and variability observed in the LSM outputs. The spatial variability of RE soil moisture standard deviation exhibits higher values in the southeastern CONUS (Fig. 6g). These standard deviation values positively correlate with precipitation, PCT_Sand, and LAI, while showing a negative correlation with temperature and distance to the coast (Fig. 6h). The same analysis method is also applied to the coefficient of variation (CV; Fig. S1), and the detailed discussion is in SI.

### Temporal wavelet analysis - comparing to *in-situ* observations

**Fig. 7:**
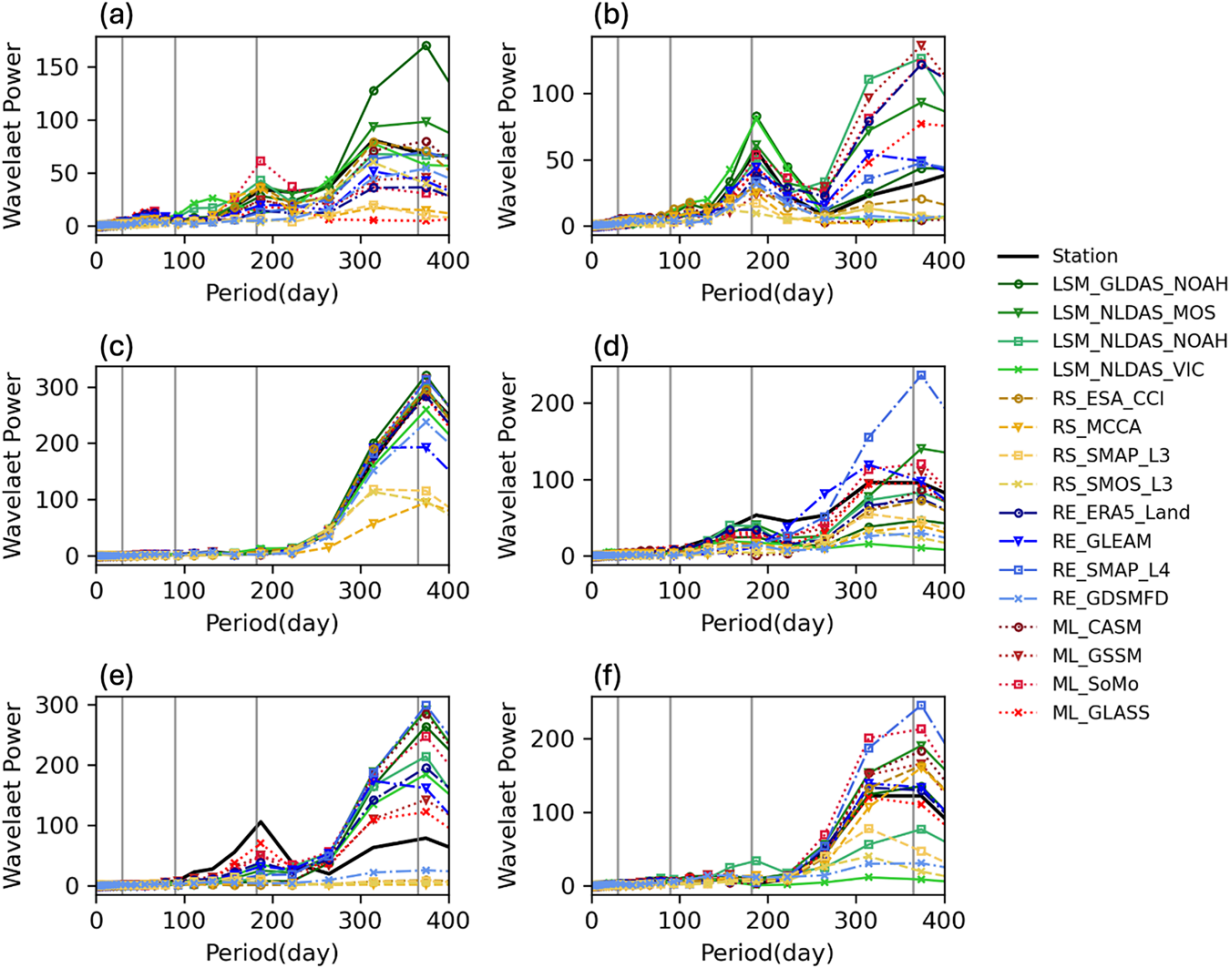
Averaged wavelet power spectrum (y-axis) versus period in days (x-axis) for the daily soil moisture (SM) time series of the station soil measurements and 16 data products at the geographic center of the six primary KGCC zones: (**a**) Arid Desert, (**b**) Arid Steppe, (**c**) Temperate DrySum, (**d**) Temperate NoDry, (**e**) Cold DrySum, and (**f**) Cold NoDry. The four vertical gray lines from left to right correspond to periods of 1 month (30 days), 3 months (90 days), 6 months (180 days) and 1 year (365 days), respectively, starting from January 2016. Since the temporal resolutions of ML_CASM, ML_RSSSM, and ML_Zheng are 3 days, 10 days, and monthly, respectively (Table 1), we did not include these three datasets for the wavelet power spectrum analysis.

Figure 7 displays a collection of wavelet power spectra comparing observed soil moisture from various sources across different climatic regions during the period 2016–2018. Each panel in the figure represents a specific KGCC. The wavelet power spectrum illustrates how the power (variance) of a time series varies with time in days (inverse of frequency). Each panel shows the period in days on the x-axis, ranging from 0 to 400 days, and the wavelet power on the y-axis, with varying scales for each climate type. The wavelet power indicates the strength of patterns or cycles within the time series data at different periods. As an illustration, there exists a peak at approximately the duration of 365 days on each plot corresponding to the annual cycle. In the plots, multiple lines represent different sources of soil moisture data: where “station” represents *in-situ* measurements from ground stations. The vertical gray lines indicate significant periodicities within the datasets, such as seasonal cycles or other important temporal features that are common across the different data sources.

The results in Fig. 7 show a variation in the wavelet power among different sources and climates, suggesting differences in how each product captures soil moisture variability. Some products show high consistency with the station data (the black line), while others diverge, especially at certain periods. This kind of analysis is useful for evaluating the performance of soil moisture estimates from different models and sources, especially in terms of their ability to capture variability over time in different climate regions.

A notable result is the systematic underestimation of wavelet power at higher frequencies (corresponding to smaller time periods or shorter time intervals) in the RS products. While the LSM products often exhibit an overestimation of power at lower frequencies (larger time periods or longer time intervals),the ML products typically offer wavelet power spectra that closely align with the spectra observed at the chosen stations. Among these ML products, ML-GSSM exhibits the highest similarity to station data in terms of spectra. This alignment could be attributed to its training or calibration using data from over ~2000 stations worldwide, potentially including some of the station data used by this study. There are specific regional disparities though, for instance, the ML products tend to overestimate seasonal variability and underestimate interannual variability in arid desert regions, while performing optimally in temperate and “Cold NoDry” zones. Regardless of the reasons for the under or overestimations, together with the deviation analyses and metrics, the results underscore the potential risks of relying on these respective products for either long-term planning or short-term decision-making, as well as early system awareness practices, associated with Earth, energy, or environmental systems, therefore, the analysis offers further insights into when to deploy each product or combine them efficiently, especially when variations across specific time scales are of interest (e.g., within daily, inter-daily, weekly, monthly, seasonal, and yearly).

## Usage Notes

This study recompiled 19 soil moisture datasets from four different sources, LSM, RE, RS, and ML, to standardized spatial and temporal resolution and coverage, adaptable to different resolutions if needed. Overall, this study provides:

1. the first comprehensive evaluation of 19 soil moisture products and substantial insights into soil moisture characteristics in terms of consistency and the distinct behaviors over CONUS.
2. a soil moisture data package that will significantly contribute to the measurement and modelling communities that intersect with soil moisture sciences.
3. a soil moisture data analysis framework that can be used as a valuable resource for similar studies in other geographic regions and over the globe, contributing to a broader understanding of soil moisture’s role in environmental processes.
4. the foundation for conducting in-depth investigations into deep-layer soil moisture dynamics, which characterize the carbon and water interactions and is essential to hydrological and agricultural applications^54,55^.

We have used some station data from the International Soil Moisture Network (ISMN)^16,17^ for additional comparison, and would point out that the ‘best-performing’ product in one region may not necessarily be the top performer in another region, where ground truthing is absent, and unfortunately extensive ground truthing covering most grids is not feasible. This study highlights:

1. the importance of understanding the behavior and limitations of soil moisture products rather than solely relying on error metrics for assessment.
2. the essential role of guidance on selecting soil moisture products based on their ability to capture relevant temporal dynamics for specific applications.
3. the needs of standardized protocols and enhancement of the spatiotemporal coverage and data quality of soil moisture.
4. the importance of understanding the strengths and limitations of each product for short-term decision-making, long-term planning, and early system awareness practices related to earth, energy, and environmental systems.

All above-mentioned efforts will enable stakeholders to make more informed decisions regarding suitability of data for specific applications and operational needs, and provides insights on when to use each product or combine them effectively, especially when focusing on variations across specific time scales.

## Supplementary information

A unified ensemble soil moisture dataset across the continental United States

## Data Availability

We have both Python and NCAR Command Language (NCL) code packages for data processing and validation. All codes used in Methods and Technical Validation are available at Zenodo, https://zenodo.org/records/14542239.
