# Supplementary material for: A unified ensemble soil moisture dataset across the continental United States
Source: Sci Data. 2025 Apr 1;12:546. doi: 10.1038/s41597-025-04657-x (PMC11961677; doi:10.1038/s41597-025-04657-x)
Supplement: Supplementary file 1 — A unified ensemble soil moisture dataset across the continental United States [file 41597_2025_4657_MOESM1_ESM.docx]

**A unified ensemble soil moisture dataset across the continental United States**

Lingcheng Li^1^, Xinming Lin^1^, Yilin Fang^1^, Z. Jason Hou^1^, L. Ruby Leung^1^,Yaoping Wang^2^, Jiafu Mao^2^, Yaping Xu^3^, Elias Massoud^4^, Mingjie Shi^1,*^

1. Pacific Northwest National Laboratory, 902 Battelle Blvd, Richland, WA 99354
2. Environmental Sciences Division and Climate Change Science Institude, Oak Ridge National Laboratory, 1 Bethel Valley Rd, Oak Ridge, TN 37830
3. Department of Environmental and Geosciences, Sam Houston State University, Huntsville, TX 77340, USA
4. Computational Earth Sciences Group, Oak Ridge National Laboratory, 1 Bethel Valley Rd, Oak Ridge, TN 37830

*Corresponding author

**Supplementary Information**

**Figures.**


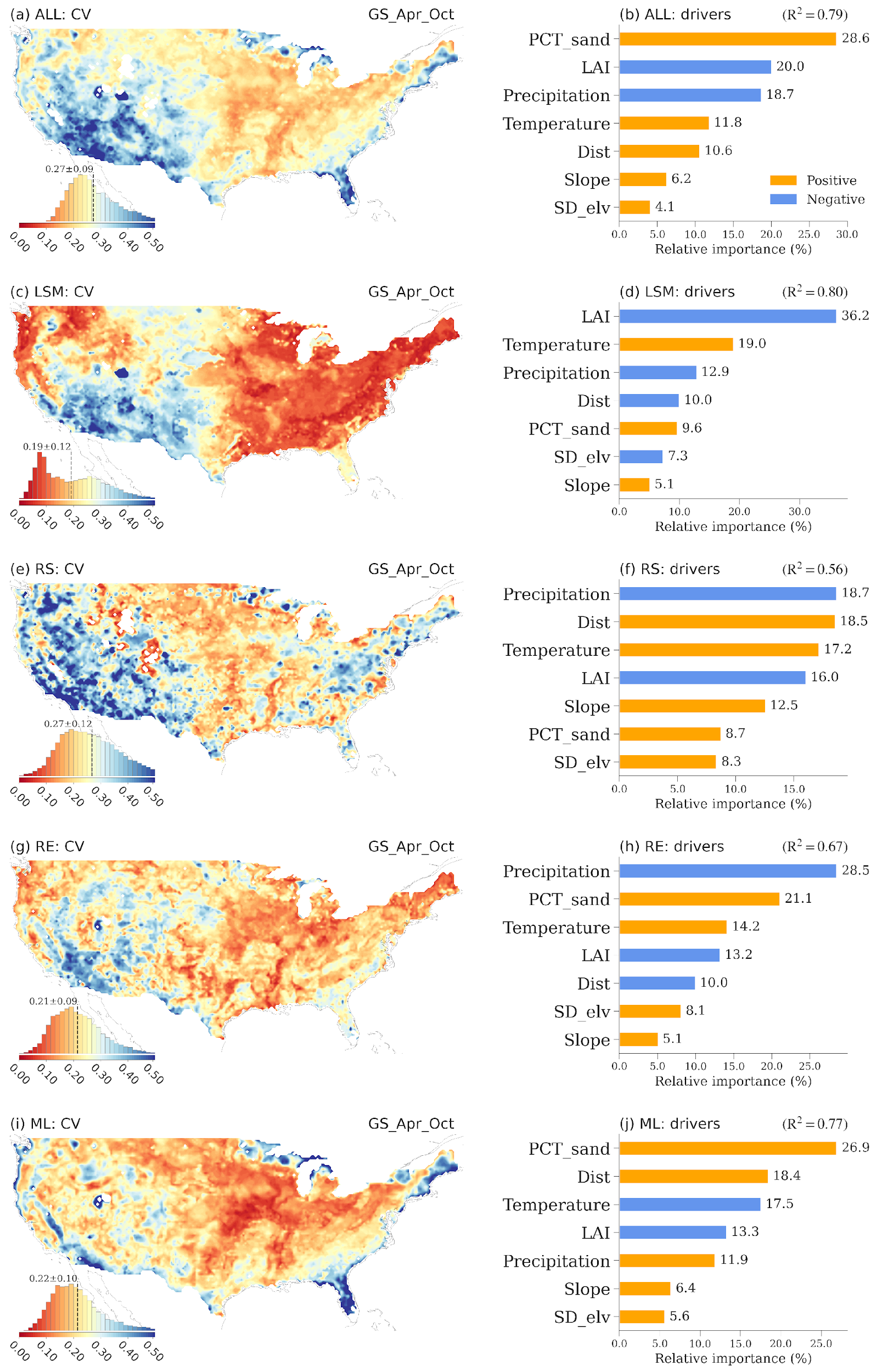


**Fig. S1**. Same to Fig. 5 but for coefficient of variation (CV). LAI represents leaf area index. Dist is the distance to the nearest coast. PCT_sand denotes the percentage of sand in the soil. Slope refers to the topographic gradient, derived from elevation data. SD_elv is the standard deviation of elevation. The details of the data sources are provided in the Methods section.


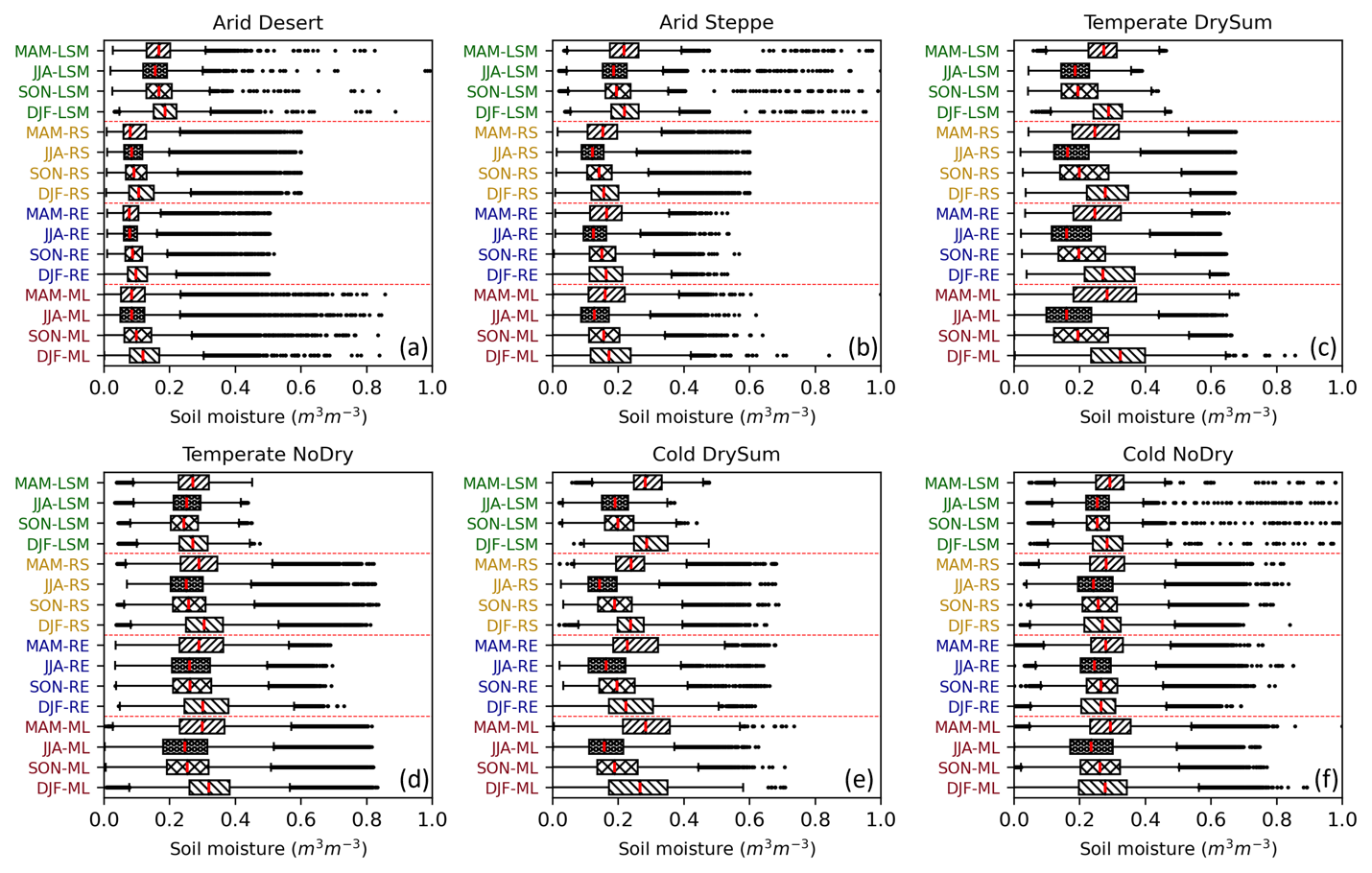


**Fig S2.** The seasonal anomalies for the four types of soil moisture over the growing season (April to October) across KGCCs. Each curve depicts the aggregated mean soil moisture anomaly for a specific type (i.e., LSM, RS, RE, or ML) calculated from gridded data across the considered KGCCs, while the shading areas show the spatial averages of SM standard deviations, providing insight into variability across data types.


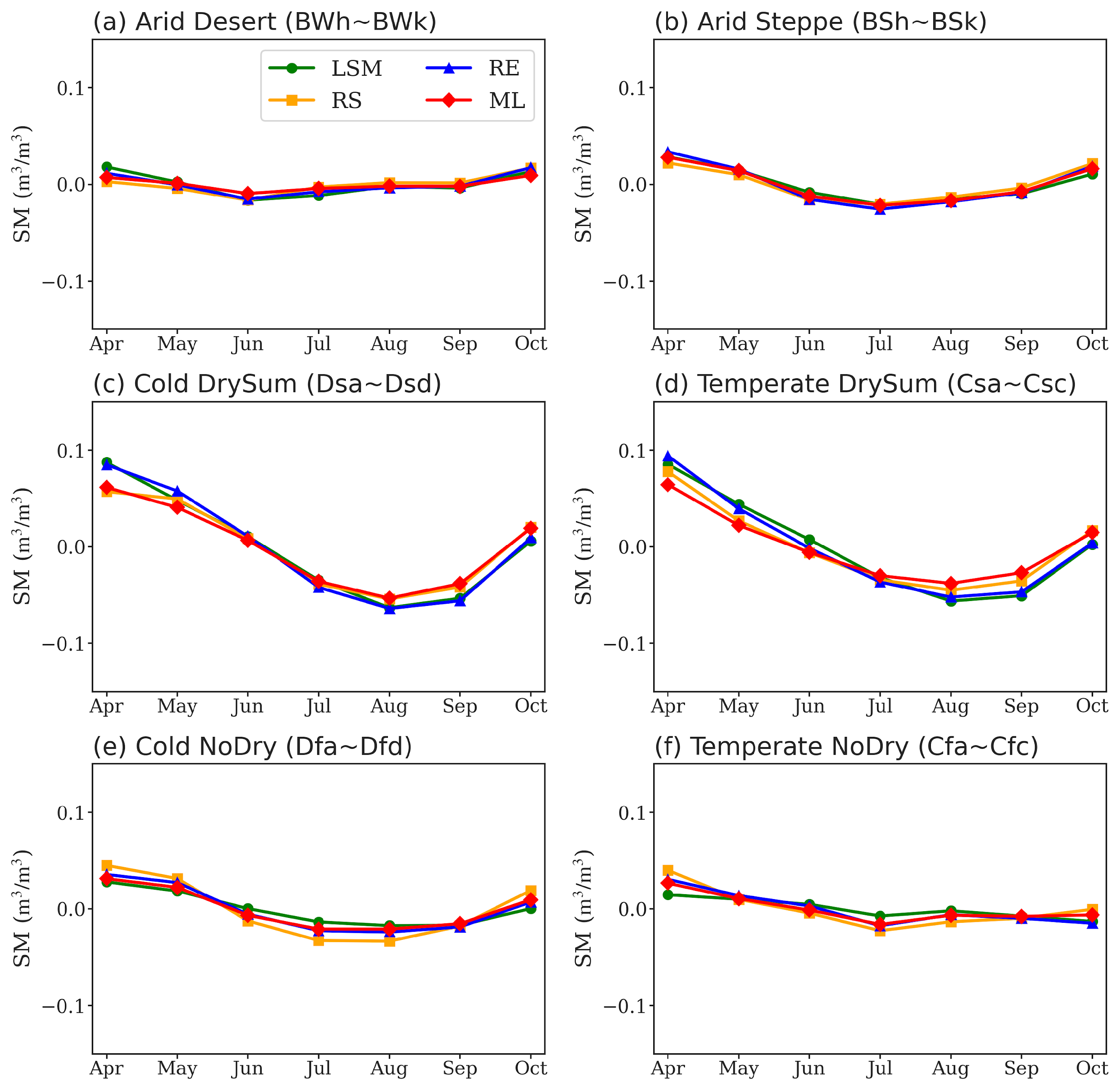


**Fig. S3.** The seasonal anomalies for the four types of soil moisture over the growing season (April to October) across KGCCs. Each curve depicts the aggregated mean soil moisture anomaly for a specific type (i.e., LSM, RS, RE, or ML) calculated from gridded data across the considered KGCCs: (a) Arid Desert, (b) Arid Steppe, (c) Cold DrySum, (d) Temperature DrySum, (e) Cold NoDry, and (f) Temperate NoDry.


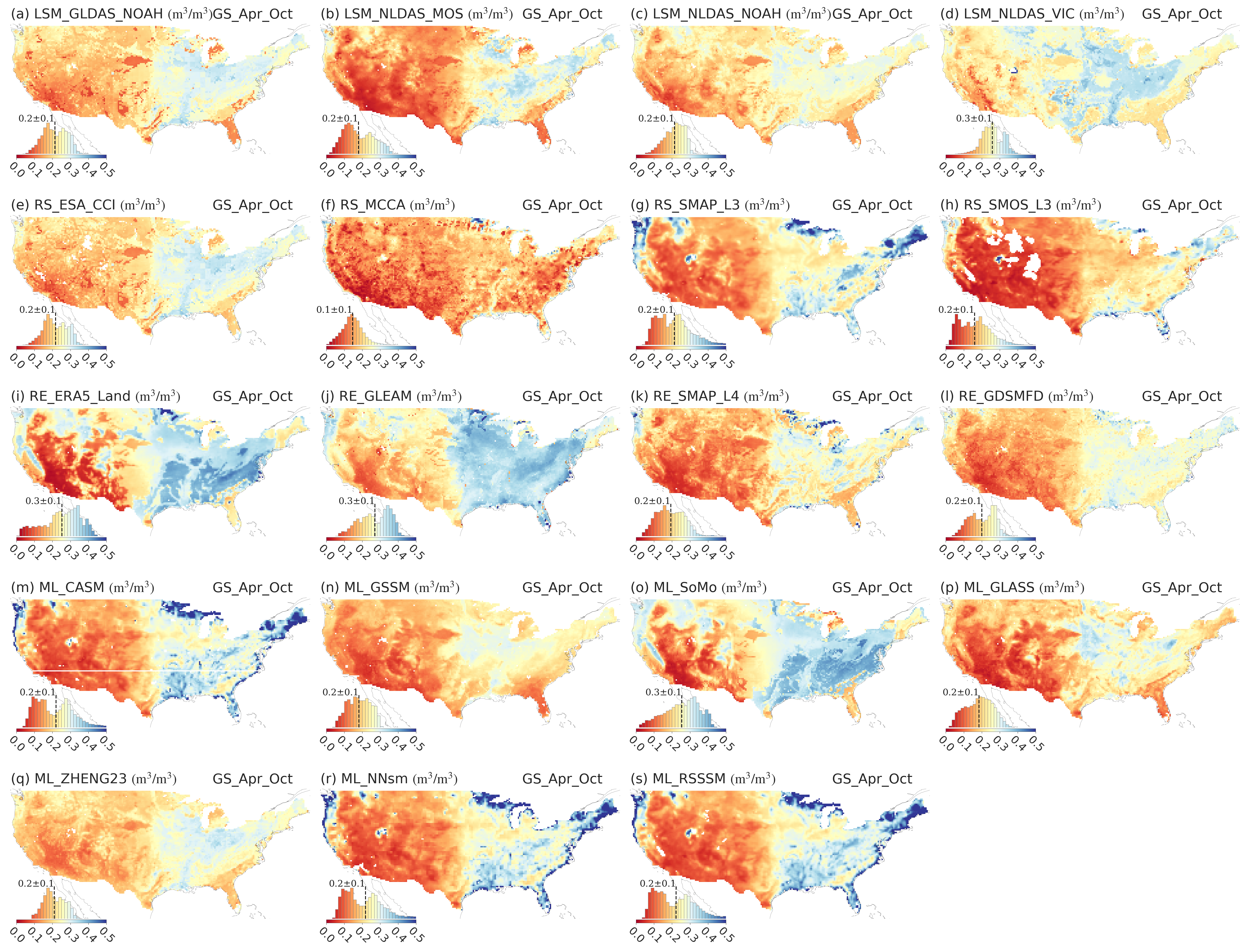


**Fig. S4.** Averaged SM during the growing season (April to October) from 2016 to 2018. Data grid cells with a minimum of 70% data record availability are used to develop the spatial maps.


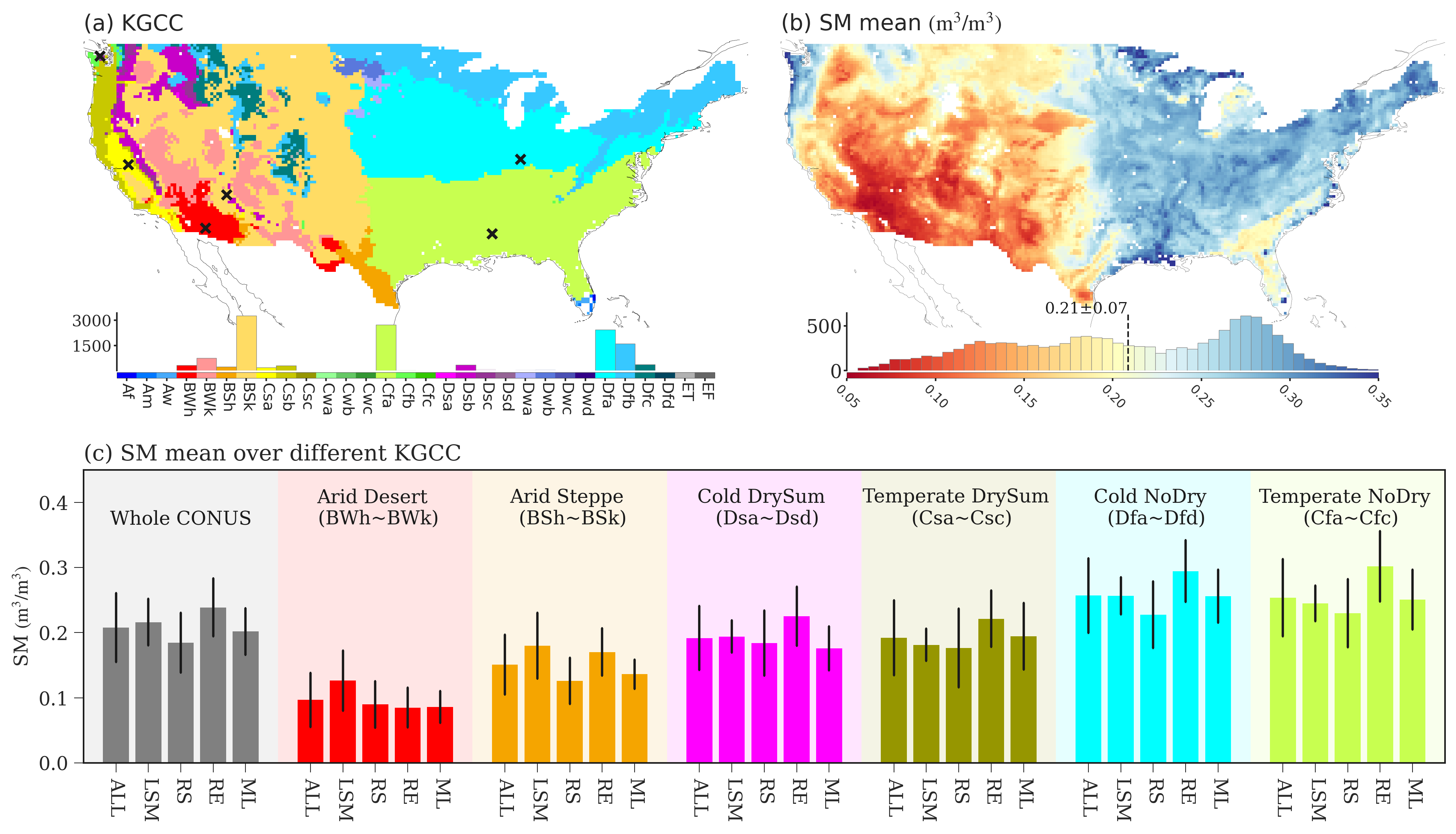


**Fig. S5.** Similar to Fig. 2, but for 16 soil moisture products with the data coverage from 2016 to 2020.


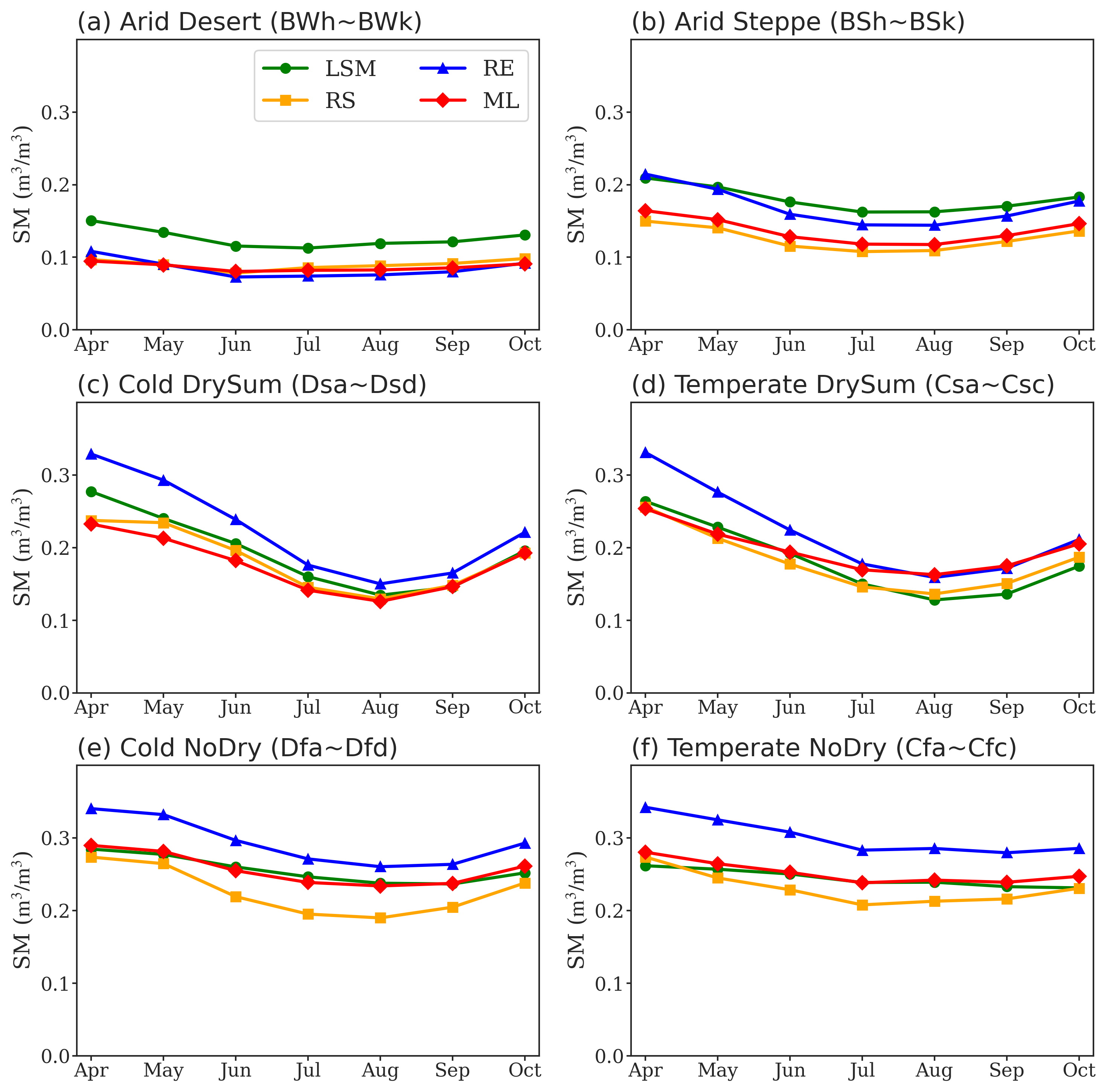


**Fig. S6.** Similar to Fig. 3, but for 16 soil moisture products with the data coverage from 2016 to 2020.


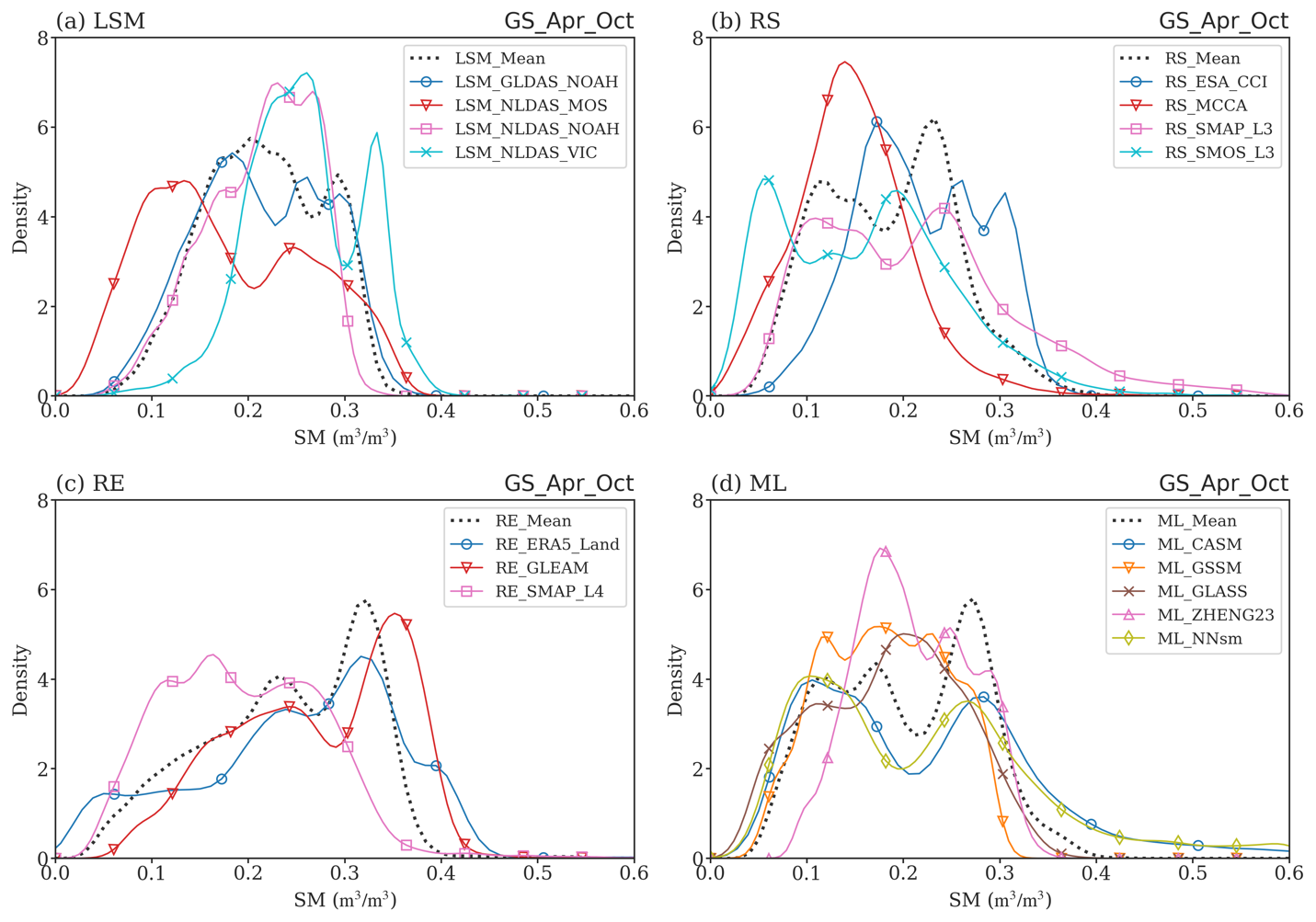


**Fig. S7.** Similar to Fig. 4, but for 16 soil moisture products with the data coverage from 2016 to 2020.


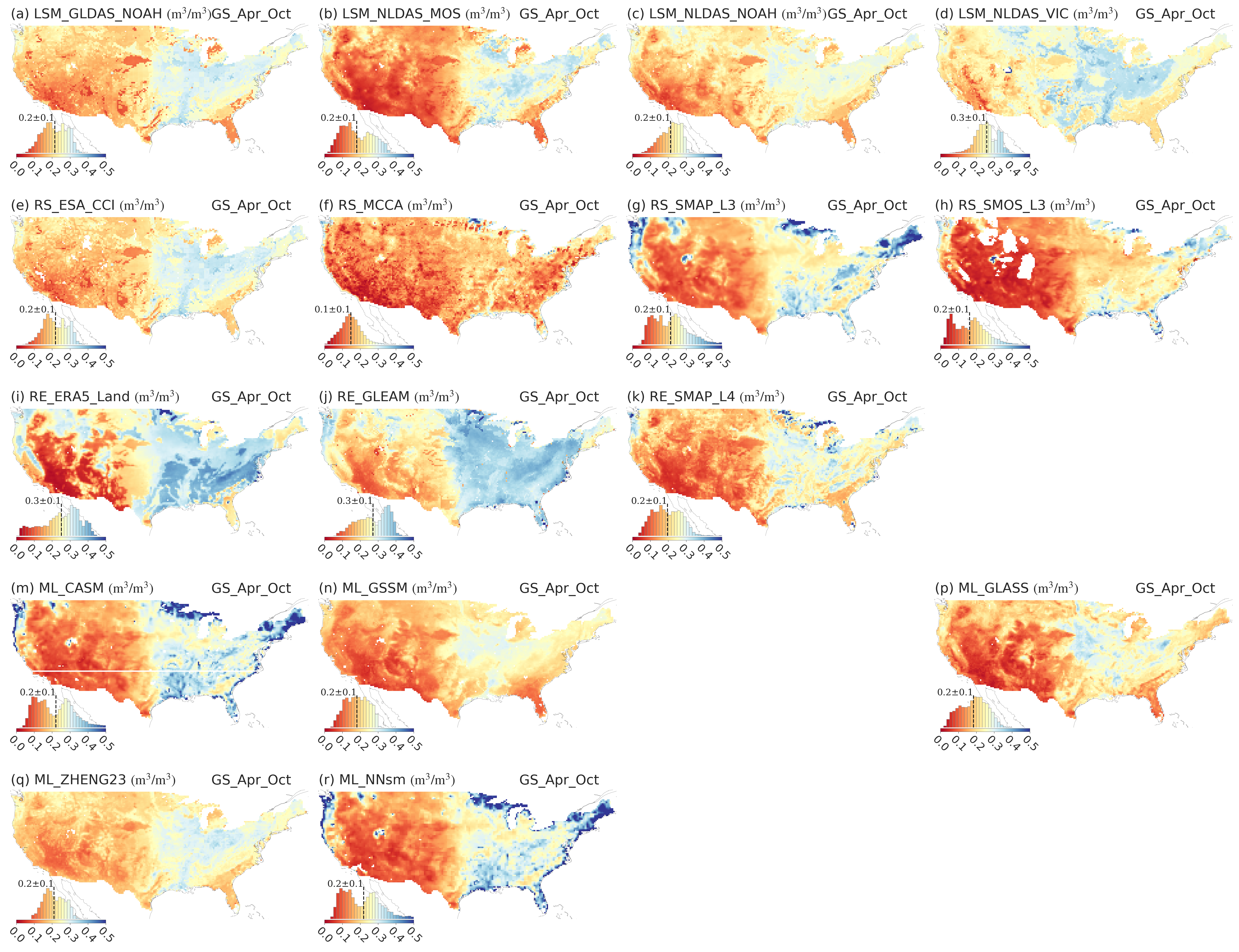


**Fig. S8.** Similar to Fig. S4, but for soil moisture products with the data coverage from 2016 to 2020. Subplots (l), (o), and (s) are omitted because these datasets do not cover the 2016–2020 period.

***Text S1. All-season soil moisture features***

The RS data retrieval can be largely affected by snow cover^31^, which generally occurs between November and March and is determined by a variety of factors, such as snowfall, topography. Thus, we perform data analysis from April to October to minimize the data uncertainties, and show the soil moisture features different data types in Fig. S2. Fig. S2 indicates that LSM is wetter than other datasets in all the “Arid” KGCCs. The “Temperate DrySum” and “Cold DrySum” KGCCs have relatively larger soil moisture seasonality than that in other KGCCs across all four data types, and this finding is consistent with that obtained from Fig. 2. The seasonalities represented by RS are similar to those represented by other data types, indicating that RS data products can be reasonably used to assess soil moisture seasonal variations over CONUS.

***Text S2. The effects of environmental factors on coefficient of variation of soil moisture products***

By using eXplainable Machine Learning and the same environmental factors discussed in Method, we also assess the importance of these factors to the coefficient of variation (CV) across different soil moisture types. As shown in Figs. 1b and S1a, the CV spatial distributions based on all the 19 datasets show that the soil moisture relative variability has large values in all the “Arid” regions and in Florida, which belongs to the “Temperate NoDry” KGCC. In all the “NoDry” KGCCs, the CV values are relatively small. For each data group, the CV spatial distributions are similar to that of the 19-data mean, showing an east–west CV value increasing trend. For LSM, the CV values in the Pacific Northwest regions are also smaller than that in the “Arid” KGCCs, implying relatively smaller data product variations represented by LSMs in this region (Fig. S1).

Percentage of sand, LAI, precipitation, and surface air temperature are the key factors influencing the CV of the 19 data products, and these factors account for 79.1% of the variability shown in the CV features. LAI, precipitation, and surface air temperature are the top contributors to the CV spatial features for LSM, RS, and, RE, and distance to coast is another important factor to the CV spatial features for LSM and RS. For ML datasets, percentage of sand, distance to coast, surface air temperature, and LAI are the top four key factors, accounting for 71% of the variability in the CV spatial map (Fig. S1). The contributions of slope and standard deviation of elevation to the CV spatial distributions are relatively minor across all data types.
